# Contamination in the Rare-Earth Element Orthophosphate Reference Samples

**DOI:** 10.6028/jres.107.056

**Published:** 2002-12-01

**Authors:** John J. Donovan, John M. Hanchar, Phillip M. Picolli, Marc D. Schrier, Lynn A. Boatner, Eugene Jarosewich

**Affiliations:** Department of Geological Sciences, The University of Oregon, Eugene, OR 97403-1272; Department of Earth and Environmental Sciences, The George Washington University, Washington, DC 20006; Department of Geology, The University of Maryland, College Park, MD 20742; Department of Chemistry, The University of California, Berkeley, CA 94720; Solid State Division, Oak Ridge National Laboratory, Oak Ridge, TN 37831; Department of Mineral Sciences, Smithsonian Institution, Washington, DC 20560

**Keywords:** EPMA, microanalysis, orthophosphates, quantitative analysis, rare earth elements, rare earth phosphates, REE, standards

## Abstract

Several of the fourteen rare-earth element (plus Sc and Y) orthophosphate standards grown at Oak Ridge National Laboratory in the 1980s and widely distributed by the Smithsonian Institution’s Department of Mineral Sciences, are significantly contaminated by Pb. The origin of this impurity is the Pb_2_P_2_O_7_ flux that is derived from the thermal decomposition of PbHPO_4_. The lead pyrophosphate flux is used to dissolve the oxide starting materials at elevated temperatures (≈1360 °C) prior to the crystal synthesis. Because these rare-earth element standards are extremely stable under the electron beam and considered homogenous, they have been of enormous value to electron probe micro-analysis (EPMA). The monoclinic, monazite structure, orthophosphates show a higher degree of Pb incorporation than the tetragonal xenotime structure, orthophosphates. This paper will attempt to describe and rationalize the extent of the Pb contamination in these otherwise excellent materials.

## 1. Introduction

Highly accurate analyses from the electron microprobe analyzer (EMPA) are only (but not solely) obtainable through the use of well-characterized and stable standards containing a major and/or known concentration of the element in question. For the rare earth elements (REE) this goal has, until recently, been elusive due to the lack of specimens exhibiting these vital properties.

The lanthanide orthophosphates, consisting of compounds with the stoichiometry LnPO_4_ where Ln represents any of the REE in the series extending from La to Lu (plus the related compounds YPO_4_ and ScPO_4_), are chemically durable and radiation resistant refractory materials. During the early 1980s a variety of single crystal rare earth orthophosphate samples were synthesized at Oak Ridge National Laboratory and the structures determined from x-ray refinements [1, 2, 3, 4, 5, and 6]. The primary purposes of these studies were varied, but they included nuclear and actinide waste disposal and scintillator material research as well as fundamental materials characterization investigations. The crystals were synthesized using a high-temperature solvent (flux-growth) technique, the details of which are available from the original papers, and a good overview of the development of these orthophosphates is discussed in Boatner and Sales [7], and references therein.

One interesting fact is that although the starting materials were carefully selected to be free from REE impurities, they were grown in a lead pyrophosphate (PbHPO_4_) flux. Pb contamination was not a concern for the original purposes of those experiments, however its presence was detected early on, and the solid state chemistry (but not the concentration) of Pb in the orthophosphate was characterized by means of electron paramagnetic resonance spectroscopy (EPR) [8]. Subsequently, these materials were investigated for possible use as standards for EPMA by the Smithsonian Institution [9], and put through a series of tests. These included homogeneity testing and a comparison to the commonly used REE doped aluminum silicate glass standards of Drake and Weill [10] using the EPMA, and a check of 10 selected REE contaminants on 7 of the compounds using instrumental neutron activation analysis. The materials appeared to be robust under electron bombardment, did not oxidize or seem hygroscopic, and no serious contamination or inhomogeneities were noted at the time and these efforts were followed by a general distribution of the material to interested parties.

In the late 1990s it was reported to one of us (JJD) that at least one investigator (E. J. Essene, University of Michigan, personal communication) had raised the issue of the role of the Pb impurity in some of the REE phosphate standards. The Pb impurity is especially significant in the CePO_4_ crystals whose black coloration is consistent with possible mixed valence (Ce^3+^ – Ce^4+^) effects—the presence of which could alter the high-temperature solid-state chemical properties and lead to an enhanced incorporation of Pb during the crystal-growth process. Subsequent investigations of the materials revealed Pb ranging in concentration from less than 0.01 mass fraction to more than 0.04 mass fraction in the CePO_4_, depending on the specific grains analyzed. It is the intent of this paper to characterize the extent of the Pb contamination in these otherwise extremely useful standards for EPMA.

## 2. Experimental Methods

Quantitative wavelength dispersive spectrometry (WDS) analyses for the REEs Sc, Y, and Pb in each of the 16 orthophosphate samples were done using a Cameca SX-51^1^ electron microprobe at 20 keV, 20 nA (2.0 × 10^−8^ A), using a 10 µm beam diameter at UC Berkeley. In addition, one of the Drake and Weill REE glasses [10], and two other REE doped calcium aluminum silicate discussed in Roeder [11] and Roeder et al. [12] were analyzed. For quantitative analyses, the K*_α_* x-ray line was used for Sc, L*_α_* lines for Y and the other REE elements, and the M*_α_* line was used for Pb. Count times were 20 s on peak and 10 s on each off-peak position except for Pb where the count times were doubled, respectively.

A complete description of the analytical setup and secondary standard accuracy for the analyzed elements (the composition of the REE phosphate primary standards in these cases had been previously adjusted for average Pb concentrations) is presented in Table 1. Secondary standards included synthetic yttrium-aluminum garnet (YAG) and alamosite (PbSiO_3_) from Tsumeb, Namibia and were assumed to be stoichiometric for Y and Pb, respectively. The Roeder REE glass S-254 [12] was assumed to have a nominal concentration (1.04 × 10^−2^ mass fraction) for La, Ce, Pr, Nd, Sm, Dy, Ho, Er, Yb, and Lu, and the Drake and Weill REE-1 glass was used based on published concentrations for Eu, Gd, Tb, and Tm [10]. For all rare-earth elements, the relative differences obtained when comparing the secondary standards to the primary standard is better than 10 % at the 0.01 mass fraction to 0.04 mass fraction concentration levels and better than 6 % in all but three cases (Pr, Sm and Lu).

The difficulty of dealing with interfering elements for REE analyses using the L*_α_* x-ray lines is painfully evident in even cursory WDS spectral scans on these samples and can only be overcome by careful and consistent application of an automatic correction scheme. Table 2 shows the REEs that interfere with the analyzed elements. These were interferences quantitatively corrected for using the iteration method of Donovan et al. [13], that is especially well suited for using large magnitude interferences for trace element determinations. For the Pb analyses, the M*_α_* line was used with a quantitative interference correction for Y (possible high order interferences from La and Tb were not observed). Standard and background intensities along with the calculated *P*/*B* (peak to background) for each line in its associated primary standard are shown in Table 3.

Under the analytical conditions which were utilized at Berkeley, the minimum detection limits for both single analyses calculated from Love and Scott [14], and for the average of 10 replicate analyses based on Goldstein et al. [15], are shown in Table 4. Minimum detection limits for 10 replicate analyses based on the actual measured standard deviation are about 3.0 × 10^−4^ mass fraction to 6.0 × 10^−4^ mass fraction for all elements in all matrices although only values for CePO_4_ or GdPO_4_ are shown in Table 4. A measured detection limit of 3 × 10^−4^ mass fraction to 6 × 10^−4^ mass fraction for the average of 10 replicates at 99 % a confidence level was typical for the REE analyses under these conditions. The Pb detection limit at a 99 % confidence level was about 4.5 × 10^−4^ mass fraction.

Another set of measurements, for the analysis of Pb homogeneity only, were also done on the same grains, but in a different area from the REE and Pb measurements done at UC Berkeley. These measurements were made for each REE orthophosphate using a JEOL 8900 Superprobe at the University of Maryland-College Park. X-ray intensities of Pb were obtained using an accelerating voltage of 20 keV, and a beam current of 150 nA. Count times were 60 s on peak, and 30 s for backgrounds on each side of the peak. Pb was analyzed using a PETH (which utilizes a smaller diameter Rowland Circle allowing for higher count rates, but has poorer wavelength resolution) crystal, and background positions of +4 mm (*L* = 173.307 mm or 5.4013 Å) and −3 mm (*L* = 166.307 mm or 5.1828 Å). Natural cerussite (PbCO_3_) from Tsumeb, Namibia, was used as a standard for Pb (0.8393 mass fraction PbO). It should be noted that although cerussite is a carbonate mineral it did not appear to degrade under the electron beam during the analyses. The Pb M*_α_* x-ray line was used for all analyses, with the exception of YPO_4_, where M*_β_* was used due to an interference from Y_l_*_γ_*_3_ on Pb M*_α_*. For these Pb homogeneity measurements, the REE and phosphate concentrations were not measured but were incorporated as stoichiometric proportions into the ZAF algorithm in order to approximately account for matrix effects. The single analysis detection limit at a 99 % confidence level for Pb under these analytical conditions was about 1.4 × 10^−4^ mass fraction Pb based on a standard count rate of 263.9 cps/nA and a background of 0.8 cps/nA measured on CePO_4_.

Measurements were done on two different sets of REE orthophosphate samples. The first set consists of material for 16 orthophosphates, including Sc and Y obtained from one of us (JMH) and mounted along with primary and secondary standards for analysis and interference corrections. These materials were mounted in a 25 mm diameter acrylic mount approximately 1.5 cm deep using a cold set epoxy and circulated to both laboratories. This sample will be referred to as the “Round Robin” mount in the discussion that follows.

The “Round Robin” mount was carefully analyzed for Pb at both Berkeley and College Park to check for inter-laboratory differences since the analytical results of trace element measurements are extremely sensitive to differences in spectrometer resolution and placement of off-peak intensity measurement positions. Homogeneity measurements were also done on this mount at College Park to check for possible Pb variations within this material itself.

Additional Pb measurements were performed at UC Berkeley on other material that was originally resident in the laboratory standard collection to check for possible inter-batch differences in Pb contamination some of the material had been produced in several runs at Oak Ridge under possibly different growth conditions. Analyses on this material will be referred to as the “Berkeley” REE mount.

## 3. Results and Discussion

### 3.1 REE Impurities in the Orthophosphate Standards

Table 5 shows the trace REE elements measured in each of the orthophosphates at UC Berkeley on the “Round Robin” mount. One can see that as stated in the original paper by Jarosewich and Boatner [9], the material is generally very pure based on quantitative results from instrumental neutron activation analysis (INAA). The only statistically significant REE contamination anomalies we observed were the presence of approximately 9 × 10^−4^ mass fraction Eu in GdPO_4_ (Jarosewich and Boatner reported 1.9 × 10^−5^ mass fraction Eu in GdPO_4_ using INAA), 1.1 × 10^−3^ mass fraction Ho and 7 × 10^−4^ mass fraction Y in the DyPO_4_ (Jarosewich and Boatner reported 2.47 × 10^−3^ Ho in DyPO_4_ using INAA, Y was not analyzed by INAA), and approximately 1.1 × 10^−3^ mass fraction Er in the TmPO_4_ (Er was not analyzed by Jarosewich and Boatner with INAA). It is difficult to obtain commercially available REE oxide materials that are completely free of other REE impurities due to the nature of the starting materials (REE-rich phosphate and carbonate minerals) that must be processed to extract individual REEs. The apparent concentration of 0.0009 ± 0.0007 mass fraction Lu in GdPO_4_ is possibly due to an interference of Gd L*_γ_*_1_ at 1.5928 Å and the 0.0006 ± 0.0005 mass fraction Dy in YbPO_4_ is possibly due to an interference of Yb Ll at 1.8946 Å and finally the 0.0004 ± 0.0003 mass fraction Yb in YPO_4_ is possibly due to an interference of Y K*_α_*_1_ (II) at 1.658 Å. No other interferences could be invoked to explain the other apparent REE concentrations shown in bold in the table.

### 3.2 Pb Impurities in the Orthophosphate Standards

The results for Pb in the last row of Table 5 reveal that Pb is present from almost 0.02 mass fraction down to about 0.005 mass fraction element in seven of the REE orthophosphates in the “Round Robin” mount (in order of decreasing concentration: CePO_4_, LaPO_4_, SmPO_4_, PrPO_4_, NdPO_4_, EuPO_4_, and GdPO_4_). The remaining REE orthophosphates did not contain Pb concentrations above the UC Berkeley detection limit of 4.5 × 10^−4^ mass fraction. These measurements consisted of a 10-point traverse on a single grain of each REE orthophosphate. Table 6 shows the Pb homogeneity measurements on the same “Round Robin” mount but performed in College Park with increased sensitivity (longer count times and higher beam currents). The two data sets agree well considering the apparent inhomogeneity of the Pb contaminated materials.

What is striking is that the Pb content varies considerably not only within each grain, but even more so from grain to grain, as seen in Table 7 where a number of Pb measurements (13–16) over the face of the four CePO_4_ grains in the “Berkeley” mount show tremendous variation between grains from about 0.015 mass fraction to 0.045 mass fraction element.

### 3.3 Crystal Structure and Pb Contamination

**Fig. 1:**
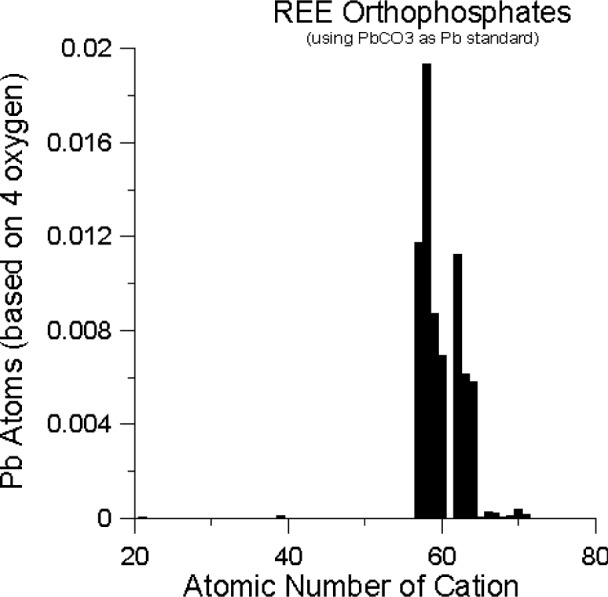
Plot of Pb atoms (based on four oxygens) versus atomic number of the REE cation. Element 61 (Pm) is unstable and the orthophosphate is not available, therefore no measurement is possible for that cation. For all others, only the monoclinic forms for the orthophosphates were observably contaminated by the Pb flux used to dissolve the starting material prior to crystal growth.

Lead is present in significant amounts only in the monoclinic, high-temperature, monazite-structure orthophosphates (LaPO_4_ through GdPO_4_), and is absent, or nearly so, in the tetragonal, xenotime-structure, compounds (TbPO_4_ through LuPO_4_ and ScPO_4_ and YPO_4_) as can be seen in Fig. 1, where Pb concentration is plotted as a function of REE atomic number. Boatner and Sales [7] showed that there is a distinct structural change (monoclinic to tetragonal) between GdPO_4_ and TbPO_4_ which suggests that the incorporation of Pb in the monazite structure, and the lack of Pb incorporation in the xenotime structure orthophosphates, is related to this change in structure. The so-called lanthanide contraction is a continuous decrease in size across the REEs, and may also play a role in this, however, there are no abrupt decreases in the trivalent ionic radii across the REE series (including from Gd to Tb). Our data suggest that the exclusion of the large (e.g., 1.29 Å in eight coordination, [16] divalent lead cation is limited by the space available in the heavy REEO_8_ (HREEO_8_) polyhedra and that the divalent Pb ion, or the trivalent HREEs, will not fit easily into the xenotime structure. For the monoclinic orthophosphates, the light REEO_9_ (LREEO_9_) polyhedra is much larger and can accommodate the divalent Pb^2+^ ion into the xenotime structure [16].

In examining the REEPO_4_ structures, it is evident that when the REE cation radius contracts beyond a certain point (empirically 1.05 Å), the REE cation becomes too small to maintain the monoclinic structure type, and the structure distorts to a lower density, lower energy, tetragonal structure type. Once this change from monoclinic to tetragonal symmetry has occurred, the divalent lead cation can no longer fit into this confined HREEO_8_ polyhedra. The tetragonal orthophosphates are all of the same structure type, with a slight contraction of unit cell volume with increasing atomic number. The same holds true for the monoclinic orthophosphates. There is a dramatic jump in the cell volumes between Gd (276 Å^3^) and Tb (292 Å^3^) with the phase change.

The tetravalent lead cation with an ionic radius of 0.94 Å [16], would appear to fit better into the tetragonal xenotime structure orthophosphates with the smaller HREE cations (1.04 Å to 0.87 Å, [16]), but significant Pb was not observed in those samples. Abraham et al. [8], did find some trivalent Pb in their EPR experiments, but other valence states of Pb such as tetravalent lead could have been present since they are not observable by means of EPR spectroscopy [8]. The flux used for crystal growth, Pb_2_P_2_O_7_, derived from the decomposition of PbHPO_4_, contains divalent lead thus, it seems more likely that the Pb was in the divalent state under the conditions of synthesis.

Characterizing the exact Pb contamination within a given orthophosphate is problematic because of the degree to which the Pb concentrations vary, not only within a single grain but also from grain to grain. For this reason it is recommended that each laboratory perform systematic x-ray mapping for Pb of their “in house” REE orthophosphates grains to determine the actual extent and variation of Pb contamination in their own mounts. As can be seen in Table 7 (e.g., grain #3), it may be that the Pb contamination is homogeneous enough that some portion or another of the material may be suitable for use as a quantitative standard for major element concentrations of the REE in question. Once the Pb concentration for a homogeneous grain is known and the position noted, the measured Pb can be proportionally subtracted from the ideal REEPO_4_ composition and entered into the laboratory’s standard compositional database for general use.

Regarding which REEPO_4_ material should be used for P as an EMPA standard, we suggest that one of the tetragonal orthophosphates should be used to minimize any nonstoichiometry introduced by Pb impurities.

## 4. Conclusions

Due to their qualities of robustness under the electron beam, resistance to oxidation, and REE purity, the REE orthophosphate standards remain a valuable set of standards for EPMA despite significant Pb contamination in at least 7 of the 16 samples examined. Of those with measurable Pb contamination, only the monoclinic CePO_4_ and possibly the LaPO_4_ and SmPO_4_ contain enough Pb to noticeably affect the stoichiometry for use as a primary standard for major element quantitative analysis (approximately 2 % to 4 % relative differences from their theoretical compositions). None of the tetragonal, xenotime structure orthophosphates (Gd-LuPO_4_ and ScPO_4_ and YPO_4_) contain appreciable Pb.

## Figures and Tables

**Table 1:** Analytical setup and measured differences between the secondary standards and the primary standard for REE quantitative analysis^a^

Element Sc K*_α_*	Spect. setup LiF (FPC-2)	Primary standard ScPO_4_ (syn.)	Secondary standard Conc in mass fraction × 10^2^	Relative diff.
YL*_α_*	PET (FPC-1)	YPO_4_ (syn.)	YAG (stoic.)	+0.368, +0.82 %
LaL*_α_*	LiF (FPC-2)	LaPO_4_ (syn.)	S-254 (1.04 nom.)	−0.020, −1.92 %
CeL*_α_*	LiF (FPC-2)	CePO_4_ (syn.)	S-254 (1.04 nom.)	−0.010, −0.95 %
PrL*_α_*	LiF (FPC-2)	PrPO_4_ (syn.)	S-254 (1.04 nom.)	−0.103, −9.95 %
NdL*_α_*	LiF (FPC-2)	NdPO_4_ (syn.)	S-254 (1.04 nom.)	−0.007, −0.70 %
SmL*_α_*	LiF (FPC-2)	SmPO_4_ (syn.)	S-254 (1.04 nom.)	−0.055, −5.27 %
EuL*_α_*	LiF (FPC-2)	EuPO_4_ (syn.)	REE-1 (3.63 pub.)	+0.069, +1.90 %
GdL*_α_*	LiF (FPC-2)	GdPO_4_ (syn.)	REE-1 (3.87 pub.)	−0.012, −0.31 %
TbL*_α_*	LiF (FPC-2)	TbPO_4_ (syn.)	REE-1 (3.78 pub.)	−0.116, −3.08 %
DyL*_α_*	LiF (FPC-2)	DyPO_4_ (syn.)	S-254 (1.04 nom.)	−0.035, −3.35 %
HoL*_α_*	LiF (FPC-2)	HoPO_4_ (syn.)	S-254 (1.04 nom.)	−0.041, −3.92 %
ErL*_α_*	LiF (FPC-2)	ErPO_4_ (syn.)	S-254 (1.04 nom.)	−0.047, −4.51 %
TmL*_α_*	LiF (FPC-2)	TmPO_4_ (syn.)	REE-1 (3.81 pub.)	−0.127, −3.33 %
YbL*_α_*	LiF (FPC-2)	YbPO_4_ (syn.)	S-254 (1.04 nom.)	−0.047, −4.53 %
LuL*_α_*	LiF (FPC-2)	LuPO_4_ (syn.)	S-254 (1.04 nom.)	−0.103, −9.94 %
PbM*_α_*	PET (FPC-1)	PbCO_3_ (Tsumeb)	PbSiO_3_ (stoic.)	+0.550, +0.75 %

a Analytical spectrometer setup (flow proportional detectors: FPC-1 indicates 1 atm P-10 and FPC-2 indicates 2 atm P-10) for REE elements (plus Sc, Y, and Pb) and results of secondary standard measurements (algebraic difference and relative difference) performed at UC Berkeley. All elements were measured at 20 keV, 20 nA (150 nA for the four grain map), 10 µm beam diameter, 20 s on-peak integration time and 10 s on each off-peak except for Pb which was counted for 40 s on-peak and 20 s on each off-peak position (240 s on-peak and 120 s on each off-peak position for the four grain map in Fig. 5). Each result shown is the average of 10 measurements.

**Table 2:** Quantitative interferences.^a^ Also listed are the wavelengths (in Å) of the x-ray lines

Element Å	On peak interferences Å
ScK*_α_* at 3.0320	ErL*_β_*_2_ (II) at 3.0284
YL*_α_* at 2.6657	LaL*_γ_*_1_ (III)at 6.4260 (not observed)
LaL*_α_* at 2.6657	NdLl (I) at 2.6766
CeL*_α_* at 2.5615	
PrL*_α_* at 2.4630	LaL*_β_*_1,4_ (I) at 2.4595, 2.4595
	SmLl (I) at 2.4826
NdL*_α_* at 2.3704	CeL*_β_*_1,4_ (I) at 2.3566, 2.3499
	PbL*_α_*_1,2_ (II) at 2.3504, 2.3732
SmL*_α_* at 2.1998	CeL*_β_*_2_ (I) at 2.2092
	PrL*_β_*_3_ (I) at 2.2175 (not observed)
EuL*_α_* at 2.1209	NdL*_β_*_3_ (I) at 2.1273
	PrL*_β_*_2_ (I) at 2.1197
GdL*_α_* at 2.0468	CeL*_γ_*_1_ (I) at 2.0489
	LaL*_γ_*_2,3_ (I) at 2.0462, 2.0415
	NdL*_β_*_2_ (I) at 2.0365
TbL*_α_* at 1.9765	LaL*_γ_*_4_ (I) at 1.9834
	PrL*_γ_*_1_ (I) at 1.9614 (not observed)
	SmL*_β_*_3_ (I) at 1.9627 (not observed)
	PbL*_β_*_1,2_ (II) at 1.9660, 1.9650 (not observed)
DyL*_α_* at 1.9088	EuL*_β_*_1,4_ (I) at 1.9207, 1.9258
	YbLl (I) at 1.8946 (possibly observed)
HoL*_α_* at 1.8450	GdL*_β_*_1,4_ (I) at 1.8472, 1.8543
	LuLl (I) at 1.8362 (not observed)
ErL*_α_* at 1.7842	TbL*_β_*_1,4_ (I) at 1.7770, 1.7867
	NdL*_γ_*_2,3_ (I) at 1.8015, 1.7968
TmL*_α_* at 1.7268	DyL*_β_*_1,4_ (I) at 1.7110, 1.7212
	GdL*_β_*_2_ (I) at 1.7457
	SmL*_γ_*_1_ (I) at 1.7275
YbL*_α_* at 1.6718	EuL*_γ_*_1_ (I) at 1.6577
	SmL*_γ_*_2_ (I) at 1.6608
	TbL*_β_*_2_ (I) at 1.6834
	YK*_α_*_1_ (II) at 1.6580 (possibly observed)
	HoL*_β_*_4_ (I) at 1.6597 (not observed)
LuL*_α_* at 1.6195	HoL*_β_*_3_ (I) at 1.6207
	DyL*_β_*_2_ (I) at 1.6241
	GdL*_γ_*_1_ (I) at 1.5928 (possibly observed)
PbM*_α_* at 5.2860	YL*_γ_*_3_ (I) at 5.2848
	LaL*_α_*_1_ (II) at 5.3326 (not observed)
	TbL*_β_*_1_ (III) at 5.3310 (not observed)

a Analyzed elements and interfering elements were quantitatively corrected by using the iteration method of Donovan et al. [12]. Many of these interferences are 1st order interferences and therefore are the same energy as the interfering line, and hence, cannot be reduced by the use of pulse height analysis (PHA). Selection of alternative (beta) lines is sometimes possible, but the resulting reduction in intensity will also reduce sensitivity.

**Table 3:** Standard peak and background intensities (linear interpolation method)^a^

Element	Peak intensity (cps/nA)	Background intensity (cps/nA)	Peak/Background
ScK*_α_*	49.3 (ScPO_4_)	0.2	246.5
YL*_α_*	68.3 (YPO_4_)	0.5	136.6
LaL*_α_*	38.5 (LaPO_4_)	0.3	128.3
CeL*_α_*	45.4 (CePO_4_)	0.5	90.8
PrL*_α_*	55.1 (PrPO_4_)	0.6	91.8
NdL*_α_*	64.9 (NdPO_4_)	0.6	108.1
SmL*_α_*	80.8 (SmPO_4_)	1.3	62.2
EuL*_α_*	89.6 (EuPO_4_)	1.1	81.5
GdL*_α_*	95.2 (GdPO_4_)	1.2	79.3
TbL*_α_*	101.9 (TbPO_4_)	1.3	78.4
DyL*_α_*	107.8 (DyPO_4_)	1.5	71.9
HoL*_α_*	113.6 (HoPO_4_)	2.2	51.6
ErL*_α_*	119.5 (ErPO_4_)	2.1	56.9
TmL*_α_*	122.9 (TmPO_4_)	2.5	49.2
YbL*_α_*	128.0 (YbPO_4_)	2.6	49.2
LuL*_α_*	131.3 (LuPO_4_)	3.4	38.6
PbM*_α_*	72.0 (PbCO_3_)	0.6	120.0

a Average peak and background intensities measured on the primary standards for the analyzed elements along with calculated peak to background ratios. Off-peak positions were based on high-resolution spectral scans of the low to high off-peak regions of each REE element and Pb in each of the REE phosphates. The purpose was to avoid off-peak interferences as much as possible.

**Table 4:** Typical single analysis and average (replicate) detection limits^a^

Element	Detection limit (single point) (.99 CL) (mass fraction × 10^2^ in CePO_4_)	Detection limit (avg. of 10) (.99 CL) (mass fraction × 10^2^ in CePO_4_)
ScK*_α_*	0.058	0.018
YL*_α_*	0.103	0.024
LaL*_α_*	0.187	0.045
CeL*_α_*	0.147 (in GdPO_4_)	0.050 (in GdPO_4_)
PrL*_α_*	0.104	0.058
NdL*_α_*	0.111	0.068
SmL*_α_*	0.103	0.042
EuL*_α_*	0.137	0.052
GdL*_α_*	0.097	0.125^b^
TbL*_α_*	0.139	0.046
DyL*_α_*	0.100	0.033
HoL*_α_*	0.140	0.042
ErL*_α_*	0.097	0.042
TmL*_α_*	0.139	0.033
YbL*_α_*	0.139	0.038
LuL*_α_*	0.142	0.043
PbM*_α_*	0.077 (in GdPO_4_)	0.045 (in GdPO_4_)

a Single point analysis detection limits in a matrix of CePO_4_ at a 99 % confidence level (CL). A GdPO_4_ matrix for Ce and Pb was used since Ce is a major element in CePO_4_ and Pb was determined to be inhomogeneous in the CePO_4_. CL and averaged detection limits for the same matrices at 99 % confidence interval based on the actual measured standard deviation of 10 measurements on each standard are reported.

b Gd is possibly present as very small, widely dispersed concentrations in the CePO_4_ which could explain this unusually high calculated detection limit (for example the calculated average detection limit for GdL*_α_* in DyPO_4_ is 0.07 mass fraction × 10^2^).

**Table 5:** Trace Pb and REE concentrations in the REEPO_4_ standards^a^ (concentrations and uncertainties in mass fraction × 10^2^)

USNM #	ScPO_4_ 168495	YPO_4_ 168499	LaPO_4_ 168490	CePO_4_ 168484	PrPO_4_ 168493	NdPO_4_ 168492	SmPO_4_ 168494	EuPO_4_ 168487
ScK*_α_*		.01 ± .01	.01 ± .02	.01 ± .01	.01 ± .01	.01 ± .01	.01 ± .01	.00 ± .00
YL*_α_*	.01 ± .02		.01 ± .01	.01 ± .01	.00 ± .01	.02 ± .03	.01 ± .02	.00 ± .00
LaL*_α_*	.01 ± .01	.02 ± .03		.00 ± .00	.03 ± .05	.02 ± .04	.03 ± .03	.01 ± .01
CeL*_α_*	.00 ± .01	.05 ± .06	.01 ± .01		.03 ± .04	.03 ± .04	.03 ± .04	.02 ± .02
PrL*_α_*	.03 ± .03	.01 ± .02	.07 ± .13^b^	.02 ± .03		.00 ± .01	.01 ± .01	.02 ± .03
NdL*_α_*	.00 ± .00	.01 ± .02	.00 ± .01	.01 ± .02	.01 ± .03		.00 ± .01	.04 ± .04
Sm*L_α_*	.02 ± .03	.01 ± .02	.01 ± .02	.02 ± .04	.01 ± .02	.00 ± .00		.01 ± .02
EuL*_α_*	.02 ± .03	.01 ± .02	.03 ± .03	.00 ± .01	.08 ± .11^b^	.03 ± .05	.03 ± .02	
GdL*_α_*	.02 ± .03	.02 ± .03	.03 ± .06	.04 ± .05	.01 ± .03	.02 ± .03	.00 ± .00	.02 ± .05
TbL*_α_*	.01 ± .01	.02 ± .03	.02 ± .03	.00 ± .01	.03 ± .04	.00 ± .00	.03 ± .05	.00 ± .01
DyL*_α_*	.03 ± .02	.02 ± .02	.03 ± .04	.02 ± .03	.00 ± .00	.00 ± .00	.00 ± .00	.02 ± .03
HoL*_α_*	.01 ± .02	.01 ± .02	.02 ± .03	.02 ± .03	.00 ± .00	.00 ± .01	.00 ± .00	.00 ± .00
ErL*_α_*	.03 ± .03	.02 ± .02	.02 ± .03	.03 ± .04	.00 ± .00	.02 ± .03	.07 ± .04	.00 ± .00
TmL*_α_*	.02 ± .02	.01 ± .02	.02 ± .02	.01 ± .02	.02 ± .03	.02 ± .02	.06 ± .07	.06 ± .06
YbL*_α_*	.00 ± .00	.03 ± .04	.02 ± .03	.04 ± .05	.04 ± .04	.03 ± .03	.02 ± .03	.02 ± .03
LuL*_α_*	.02 ± .03	.01 ± .03	.01 ± .02	.03 ± .04	.02 ± .03	.04 ± .03	.00 ± .00	.00 ± .01
PbM*_α_*	.00 ± .00	.01 ± .01	1.05 ± .17	1.68 ± .07	.77 ± .04	.60 ± .03	.99 ± .07	.52 ± .06

	GdPO_4_ 168488	TbPO_4_ 168496	DyPO_4_ 168485	HoPO_4_ 168489	ErPO_4_ 168486	TmPO_4_ 168497	YbPO_4_ 168498	LuPO_4_ 168491
ScK*_α_*	.01 ± .01	.00 ± .00	.01 ± .01	.01 ± .02	.01 ± .02	.00 ± .00	.00 ± .01	.01 ± .02
YL*_α_*	.01 ± .02	.01 ± .02	.07 ± .05	.02 ± .03	.02 ± .03	.01 ± .01	.04 ± .03	.03 ± .03
LaL*_α_*	.02 ± .04	.01 ± .02	.02 ± .04	.03 ± .04	.01 ± .02	.01 ± .01	.03 ± .05	.02 ± .03
CeL*_α_*	.03 ± .04	.01 ± .02	.02 ± .02	.01 ± .03	.03 ± .04	.01 ± .01	.01 ± .02	.01 ± .02
PrL*_α_*	.01 ± .02	.01 ± .03	.01 ± .02	.02 ± .03	.02 ± .04	.01 ± .03	.02 ± .05	.01 ± .02
NdL*_α_*	.01 ± .02	.02 ± .03	.01 ± .02	.01 ± .03	.02 ± .04	.03 ± .04	.00 ± .00	.03 ± .04
SmL*_α_*	.02 ± .03	.00 ± .01	.03 ± .04	.00 ± .01	.02 ± .02	.03 ± .03	.03 ± .03	.02 ± .03
EuL*_α_*	.09 ± .06	.03 ± .03	.00 ± .01	.00 ± .01	.01 ± .03	.01 ± .01	.01 ± .02	.01 ± .01
GdL*_α_*		.01 ± .02	.01 ± .02	.00 ± .01	.00 ± .00	.02 ± .02	.02 ± .03	.01 ± .01
TbL*_α_*	.01 ± .02		.02 ± .03	.01 ± .01	.00 ± .00	.02 ± .03	.02 ± .03	.01 ± .03
DyL*_α_*	.00 ± .00	.00 ± .00		.00 ± .00	.02 ± .04	.00 ± .00	.06 ± .05	.01 ± .03
HoL*_α_*	.14 ± .21^b^	.01 ± .02	.11 ± .06		.01 ± .01	.01 ± .03	.02 ± .04	.03 ± .03
ErL*_α_*	.00 ± .00	.05 ± .09	.01 ± .02	.00 ± .00		.11 ± .07	.02 ± .02	.00 ± .01
TmL*_α_*	.01 ± .02	.00 ± .00	.03 ± .05	.03 ± .03	.00 ± .00		.00 ± .01	.04 ± .04
YbL*_α_*	.01 ± .01	.02 ± .03	.00 ± .01	.00 ± .00	.03 ± .04	.00 ± .00		.00 ± .00
LuL*_α_*	.09 ± .07	.02 ± .03	.02 ± .05	.05 ± .07	.00 ± .00	.01 ± .04	.00 ± .00	
PbM*_α_*	.49 ± .07	.02 ± .02	.02 ± .03	.02 ± .03	.02 ± .02	.01 ± .02	.02 ± .03	.04 ± .04

a Average trace analyses of REE elements plus Sc, Y, and Pb for the USNM REE phosphates in the “Round Robin” mount measured at Berkeley. The quoted uncertainty is the measured one standard deviation value for 10 measurements.

b Large magnitude interference corrections resulting in increasing uncertainty at trace levels. The apparent concentrations and large standard deviations for these three cases could be greatly reduced by using longer acquisition times on the unknown and the standard used for the interference correction.

**Table 6:** Pb (mass fraction × 10^2^) in the “round robin” mount measured in College Park^a^

	ScPO_4_	YPO_4_^b^	LaPO_4_	CePO_4_	PrPO_4_	NdPO_4_	SmPO_4_	EuPO_4_
PbM*_α_*	.00 ± .00	.00 ± .00	.90 ± .32	1.90 ± .07	.92 ± .04	.86 ± .17	.86 ± .13	.64 ± .16

	GdPO_4_	TbPO_4_	DyPO_4_	HoPO_4_	ErPO_4_	TmPO_4_	YbPO_4_	LuPO_4_
PbM*_α_*	.39 ± .16	.00 ± .00	.00 ± .00	.00 ± .00	.00 ± .00	.00 ± .00	.00 ± .00	.00 ± .00

a Averaged mass fraction × 10^2^ results of Pb contamination measurements performed in College Park on the “round robin” mount. The mass fraction detection limit (99 % confidence level) was approximately 140 × 10^−6^. Note that the measured Pb standard deviations for the uncontaminated materials are significantly smaller than the measurements performed at Berkeley. These results are due to the increased beam current and counting time used at College Park.

b PbM*_β_* was used to avoid the Y_l_*_γ_*_3_ line.

**Table 7:** Pb grain to grain variation within the CePO_4_ material in the “Berkeley” mount^a^

	Average (concentrations in mass fraction × 10^2^)	Standard deviation	Minimum	Maximum
Grain #1	2.68	0.45	2.04	3.47
Grain #2	2.55	0.16	2.33	2.83
Grain #3	1.54	0.04	1.48	1.59
Grain #4	3.64	0.46	3.08	4.50

a Average and standard deviations (13-16 points over the face of each grain) of four grains from the “Berkeley” mount mapped in Fig. 1 in elemental mass fraction × 10^2^. Analytical conditions were 20 keV, 150 nA, and a 10 µm diameter beam. Each analysis is the average of 13 to 16 measurements distributed over the face of each grain. Only grain #3 was relatively homogeneous in Pb.

## Footnotes

1 NIST disclaimer: Certain commercial equipment, instruments, or materials are identified in this paper to foster understanding. Such identification does not imply recommendation or endorsement by the National Institute of Standards and Technology, nor does it imply that the materials or equipment identified are necessarily the best available for the purpose.
